# Dynamic Changes in Heart Rate Variability and Nasal Airflow Resistance during Nasal Allergen Provocation Test

**DOI:** 10.1155/2016/1245418

**Published:** 2016-02-14

**Authors:** Tiina M. Seppänen, Olli-Pekka Alho, Tapio Seppänen

**Affiliations:** ^1^Center for Machine Vision and Signal Analysis, University of Oulu, Oulu, Finland; ^2^Medical Research Center Oulu, Oulu University Hospital and University of Oulu, Oulu, Finland; ^3^Department of Otorhinolaryngology, Oulu University Hospital, Oulu, Finland; ^4^Research Unit of Otorhinolaryngology and Ophthalmology, University of Oulu, Oulu, Finland

## Abstract

Allergic rhinitis is a major chronic respiratory disease and an immunoneuronal disorder. We aimed at providing further knowledge on the function of the neural system in nasal allergic reaction. Here, a method to assess simultaneously the nasal airflow resistance and the underlying function of autonomic nervous system (ANS) is presented and used during the nasal provocation of allergic and nonallergic subjects. Continuous nasal airflow resistance and spectral heart rate variability parameters show in detail the timing and intensity differences in subjects' reactions. After the provocation, the nasal airflow resistance of allergic subjects showed a positive trend, whereas LF/HF (Low Frequency/High Frequency) ratio and LF power showed a negative trend. This could imply a gradual sympathetic withdrawal in allergic subjects after the allergen provocation. The groups differed significantly by these physiological descriptors. The proposed method opens entirely new opportunities to research accurately concomitant changes in nasal breathing function and ANS.

## 1. Introduction

Allergic rhinitis is a major global health problem affecting about 10–20% of the population in all countries, ethnic groups, and ages. Using a conservative estimate, it occurs in over 500 million people and its prevalence is increasing in most countries. It is a major chronic respiratory disease that weakens quality of life, school/work productivity, and performance. Additionally, it is a substantial economic burden to societies. Allergic rhinitis is an inheritable systemic inflammatory condition which links, for example, with asthma [1]. However, it is very commonly underdiagnosed and undertreated, because of which specific programs and guidelines on the problem have been released, for example, by World Health Organization and European Union [1–3].

Allergic rhinitis is diagnosed when specific antigens are detected in the blood and the patient has symptoms that correspond with exposure to the sensitizing allergen. It is characterized by one or more symptoms, including nasal obstruction, rhinorrhea, sneezing, nasal itching, and eye irritation [4]. Nasal provocation tests can be used to verify the presence of allergy. Subjects are challenged with the suspected allergen and changes in the symptoms are recorded and possibly the amount of secretions and the respiratory function of the nose are measured. Nasal provocation tests are the standard procedure to evaluate the clinical response of the nasal mucosa to allergens with high specificity and sensitivity [5]. It is of special relevance in the detection of patient with local allergic rhinitis and has been used for the clinical monitoring of antiallergic drugs and allergen-specific immunotherapy and also provides information on the etiology of occupational respiratory diseases of allergic origin. To rule out nonspecific nasal hyperreactivity, nasal provocation tests are usually started with challenging the nasal mucosa with a control solution.

Nasal breathing function is difficult to quantify directly by the patient's own comprehension, history, and clinical examination, which calls for objective measurement methods. Examples of these include acoustic rhinometry, peak nasal inspiratory flow measurement (PNIF), and rhinomanometry [6, 7]. Acoustic rhinometry assesses nasal geometry by measuring cross-sectional area of the nose as a function of the distance from the nostril. PNIF is a method that measures the nasal airflow during maximal forced nasal inspiration. Rhinomanometer measures the simultaneous nasal pressure and airflow from the values of which nasal airflow resistance is determined [8]. The resistance is characteristically described as a number that derives from one or more breathing cycles of data. Additionally, nasal cavities are measured one at a time and the total nasal resistance is calculated based on unilateral resistances. Thus, it is not possible to get accurate instantaneous values. In practice, there are several minutes between the consecutive resistance values that a rhinomanometer can provide. In nasal provocation tests, the major response to measure is the rise in nasal airflow resistance, which can be rapid (seconds or minutes), and the timing differs in different individuals. This makes it difficult to be detected with the above-mentioned methods. One possibility is to determine the resistance with the rhinomanometer in certain time intervals, but this cannot be done very rapidly and it has been indicated to give inconsistent and variable results with low reproducibility [9–11]. Thus, there is a need for a measurement giving accurate and continuous measurement data about the nasal breathing function.

The nose is armed with a complex nervous system that includes parasympathetic, sympathetic, and sensory nerves. Through the nervous system, the nose communicates with the cardiovascular apparatus, the lungs, and the gastrointestinal tract. Through neural interactions, events that are initiated in the nose can be transmitted to other organs and vice versa. The allergic response comprises changes at all levels of the neural arc: central nervous system integration, sensory nerve function, and autonomic/enteric neuroeffector cell function. Central sensitization can lead to the parasympathetic dominance which originated from the central nervous system. Neural hyperresponsiveness is believed to play a pivotal role in the allergic rhinitis. It would appear that the nervous system itself is altered and is rendered hyperactive in many allergic patients [12, 13].

Heart rate variability (HRV) analysis is an indirect noninvasive way to assess autonomic nervous system (ANS) modulation. It quantifies the degree of fluctuation of the beat-to-beat differences in cardiac rhythm [14]. The interactions between respiration, heart rate, and blood pressure fluctuations, which reflect the cardiovascular and cardiorespiratory couplings, are considered to be of paramount importance for the study of the ANS [15]. Relatively few studies have examined the association between the allergic rhinitis and autonomic nervous system, especially using HRV analysis. Lan et al. evaluated the effect of position on the autonomic nervous system of allergic and control volunteers by HRV analysis. They concluded that patients with allergic rhinitis may have poor sympathetic modulation in the sitting position [16]. Taşcilar et al. measured 24-hour ECG recordings from pediatric allergic rhinitis patients with allergic rhinitis and healthy controls. Their HRV analysis' results implied sympathetic withdrawal and parasympathetic predominance in pediatric allergic rhinitis patients with allergic rhinitis [17]. Yokusoglu et al. in their turn measured 24-hour ambulatory ECG recordings from adults with allergic rhinitis and healthy controls. They found out that HRV indices predicting the parasympathetic predominance were increased in patients with allergic rhinitis [18].

Recently, we presented a novel method to assess nasal airflow resistance continuously and accurately [19]. The pressure signal is measured with a thin nasopharyngeal catheter inserted into the nasopharynx and the airflow signal with the rib cage and abdominal effort belts calibrated with our new method [20]. The resistance is calculated for each signal sample at any sampling frequency. This makes it possible to discover rapid changes in nasal airflow resistance, which is essential, for example, during provocation tests. In this study, the continuous nasal airflow resistance is produced using the above-mentioned methods and dynamically changing autonomic nervous system parameters are computed to study their simultaneous changes during an allergen provocation test. To our knowledge, this kind of study has not been done before, apparently because it has not been possible to produce nasal airflow resistance curves in this precision. Our objectives are to examine, firstly, whether there are associations between the dynamic reactions of nasal airflow resistance and autonomic nervous system parameters during the allergic reaction and, secondly, whether there are differences between the birch pollen allergic and the nonallergic groups.

## 2. Methods

### 2.1. Materials

An experimental study design was used. The study protocol was approved by the Regional Ethics Committee of the Northern Ostrobothnia Hospital District. In Finland, the birch pollen is one of the most common causes of the intermittent seasonal allergic symptoms; for this very reason, it was chosen as a provocation substance. For this preliminary study, ten (three females) birch pollen allergic and ten (three females) nonbirch pollen allergic adult volunteers were recruited. The diagnostic criteria for birch pollen allergic rhinitis were evidence of sensitization to birch pollen measured by the presence of allergen-specific IgE in the serum and a history of nasal symptoms during the birch pollen season [21]. All the volunteers gave written informed consent for participation in the study. The mean (SD) age of the allergic and nonallergic subjects was 24 (1) and 24 (3) years, respectively. The subjects had to be nonpregnant and free of surgical operations of nose, brain circulatory disorders, and heart diseases. Medication that affects the nose was not allowed to be used during a specific time period before the measurement. The subjects had to be free of any acute respiratory symptoms during the prior two weeks to the measurement. Before measurement, subjects refrained from having a smoke for at least four hours and heavy meal, caffeine, or other stimulants for at least two hours.

Measurements were made outside the birch pollen season. The subjects were examined by an ear, nose, and throat specialist. Before measurements, the specific IgE for birch pollen was determined from blood for all of them. Based on the blood samples, antibody levels of allergic subjects varied from moderate to very high.

ECG, pressure, and respiratory effort belt signals were recorded with the commercial polygraphic recorder (Embletta Gold, Denver, Colorado, USA). It had inductive respiratory effort belts for rib cage and abdomen with the sampling rate of 50 Hz. The sampling rates of pressure and ECG were 50 Hz and 200 Hz, respectively. For calibrating the rib cage and abdominal effort belt signals, simultaneous respiratory airflow signal was recorded with a spirometer (SpiroStar USB M9460, Medikro Oy, Kuopio, Finland) with the sampling rate of 100 Hz.

### 2.2. Challenge Protocol

A water-based immunologically standardized commercial 1 : 10000 SQU/mL birch extract (Allergologisk Laboratorium A/S, Copenhagen, Denmark) was used in the provocation. The diluent solution (Allergologisk Laboratorium A/S, Copenhagen, Denmark) of the allergen extract was used as a control solution. Both solutions were administered bilaterally into the nasal cavities by pump spray.

**Figure 1:**
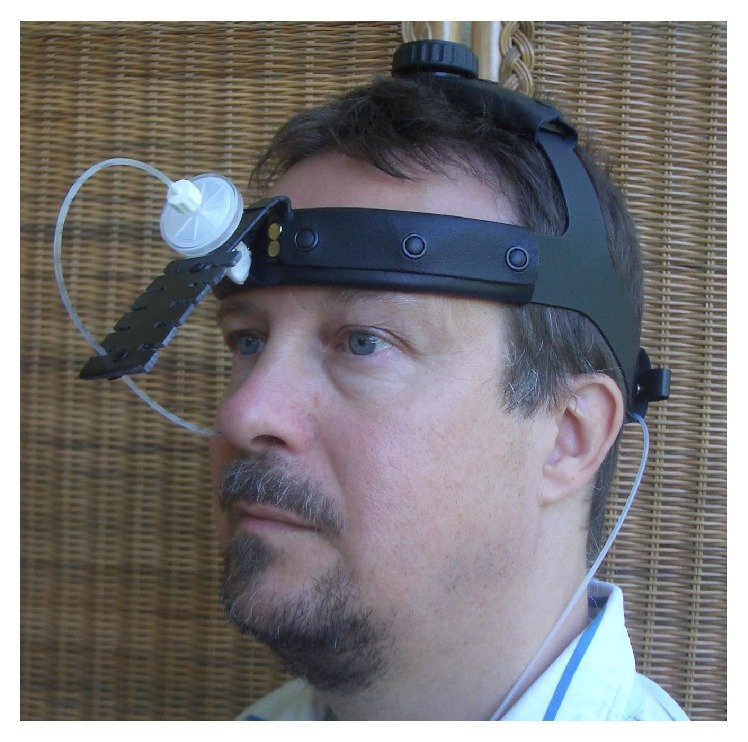
Measurement setup of nasal pressure signal with nasopharyngeal catheter.

At the beginning of measurement, the rib cage effort belt was placed on the xiphoid process and the abdominal effort belt was placed near the umbilicus. Next, the subjects sat peacefully for a period of 30 min prior to the measurement to adapt themselves to the environment and to allow heart rate and blood pressure to stabilize. During the actual measurements, they were instructed to sit in back upright position avoiding speaking and movements. First, respiratory effort belt signals were recorded along with the spirometer signal for one minute. The data was used for calibrating the rib cage and abdominal effort belt signals to flow signals, as described in Section 2.3. After calibration, the spirometer was removed from the subject and a thin catheter (diameter: 1 mm) was inserted 8 cm deep along the floor of nasal cavity into the nasopharynx, with the tip of the catheter lying 1 cm anterior from the back wall of nasopharynx.  Figure 1 shows the setup for the nasal pressure measurement with the catheter.

To inhibit the nasal secrete blocking, the nasal catheter air was blown with the syringe through the catheter before each measurement protocol phase and every time that the catheter blocking was detected.

The measurement protocol consisted of the three phases. At the first protocol phase, the ECG, pressure, and respiratory effort belt signals were recorded for 10 min to get the baseline situation. At the second phase, to rule out the nonspecific nasal hyperreactivity, the nasal mucosa was challenged with a control solution. It was sprayed carefully on the anterior mucosa of both nasal cavities, after which the ECG, pressure, and respiratory effort belt signals were recorded for 5 min. At the third phase, the birch pollen solution was sprayed carefully on the anterior nasal mucosa of both nasal cavities. After that, the ECG, pressure, and respiratory effort belt signals were recorded for 20 min. After spraying the solution, the recording was initiated as soon as possible but first waiting for the immediate reactions such as sneezing and snuffling to settle.

Before analysis, all the signals were validated manually by using specially made visualization software. All the detected disturbances, which originated, for example, from moving, snuffling, sneezing, and blowing of air with the syringe through the catheter, were deleted from signals before analysis. Additionally, special care was taken to maintain the correct synchrony between the signals.

### 2.3. Calculation of the Continuous Nasal Airflow Resistance

**Figure 2:**
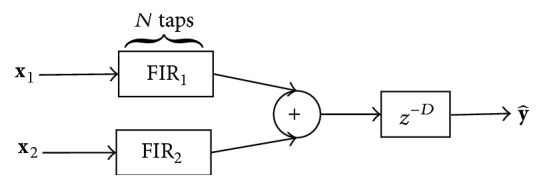
Improved respiratory effort belt calibration method.

In this study, we use our previously published novel method to estimate continuous nasal airflow resistance using pressure signal from the nasopharyngeal catheter inserted transnasally into the nasopharynx and calibrated rib cage and abdominal effort belt signals [19]. Recently, we published an improved respiratory effort belt calibration method [20], which reduces waveform errors considerably and is robust to breathing style changes. It is based on an optimally trained FIR (Finite Impulse Response) filter bank constructed as a MISO (Multiple-Input Single-Output) system between respiratory effort belt signals and the spirometer and a delay element (*z* ^−*D*^); see Figure 2. The method extends the traditional multiple linear regression method by using a number *N* of consecutive signal samples and linear filtering for each prediction.

With the respiratory effort belts used in this study, the following realization of the filter bank was used:

(1) $$\begin{array}{c} y\left[\;j\;\right]=\alpha_{1}^{T}x_{1}\left[\;j\;\right]+\alpha_{2}^{T}x_{2}\left[\;j\;\right]+\varepsilon \left[\;j\;\right], \end{array}$$

where **α**_1_^*T*^ and **α**_2_^*T*^ denote the *N* tap coefficients of filters FIR_1_ and FIR_2_, respectively. Superscript *T* indicates the matrix transpose in the formula, and *j* denotes the time index of the signals. Variable *y* indicates the respiratory airflow from spirometer. Vectors **x**_1_ and **x**_2_ include *N* consecutive signal samples from the rib cage signal and abdomen signal, respectively: **x**_*k*_[*j*] = [*x*_*k*_[*j*], *x*_*k*_[*j* − 1],…,*x*_*k*_[*j* − *N* + 1]]^*T*^, where *k* = 1,2 and *j* = *N*,…, *n*. With this measurement data, the variable *n* was 3000, which was the number of observations used in the calibration.

During the calibration, tap coefficients **α**_1_^*T*^ and **α**_2_^*T*^ are estimated with the method of least squares from the available data. The least-squares estimator of the parameter vector **α** = [**α**_1_^*T*^, **α**_2_^*T*^]^*T*^ is given by

(2) $$\begin{array}{c} \hat{\alpha }={\left(\;X^{T}X\;\right)}^{-1}X^{T}y, \end{array}$$

where **X** is (*n* − *N* + 1)×(2 × *N*) matrix formed from the vectors **x**_1_ and **x**_2_:

(3) $$\begin{array}{c} X=\left[\begin{array}{cc} x_{1}^{T}\left[N\right] & x_{2}^{T}\left[N\right]\\ x_{1}^{T}\left[N+1\right] & x_{2}^{T}\left[N+1\right]\\ \vdots & \vdots \\ x_{1}^{T}\left[n\right] & x_{2}^{T}\left[n\right] \end{array}\right]. \end{array}$$

The length of the vector $\hat{\alpha }$ is 2 × *N*. Thus, the flow signal predicted from the rib cage and abdominal effort belt signals through the FIR filter bank is

(4) $$\begin{array}{c} \hat{y}=X\hat{\alpha }. \end{array}$$

In our previous study [22], the 0.3 sec length of FIR filters was found to produce the best airflow prediction with the same respiratory effort belts. Thus, we used *N* value of 16 in this study.

**Figure 3:**
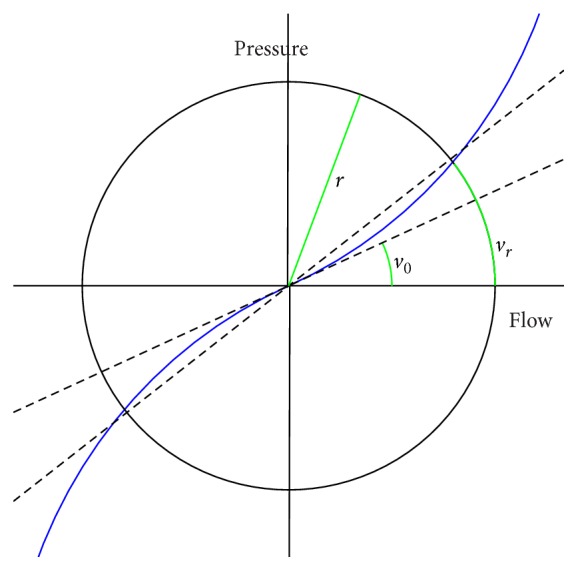
A graph of Broms model of resistance.

Mathematical model of Broms et al. [23] offers parametric means to describe the nonlinear pressure/flow relationship; see Figure 3. In the model, the pressure/flow relationship is established as follows:

(5) $$\begin{array}{c} v_{r}=v_{0}+cr, \end{array}$$

where *v*_*r*_ is the angle with radius *r*, *v*_0_ is the angle in the origin, and *c* is the curvature parameter. The resistance in radius *r*, denoted by *R*_*r*_, is given by

(6) $$\begin{array}{c} R_{r}=x\tan v_{r}. \end{array}$$

The constant *x* is a normalization factor defined in [19].

However, the Broms model expects the data to be stationary, while the nasal system is nonstationary. Our method to estimate continuous nasal airflow resistance is a least-mean-square (LMS) extension to the Broms model [23] so that it can be used for calculating a continuous nasal airflow resistance through model adaptation to time-varying characteristics of the nasal functioning. In the extended model, an instantaneous nasal airflow resistance can be calculated after estimating the model parameters at each time instant from the input signals. The normalized [19] pressure signal, *P*′, and the estimate of flow signal, *F*′, are the LMS filter inputs. The filter length of one time sample has proven to be sufficient. The update formulas of parameters *v*_0_ and *c* are

(7) v0i+1=v0i+μivri−v0i+ciri,ci+1=ci+μirivri−v0i+ciri,

where

(8) vri=tan−1⁡P′iF′i,ri=P′2i+F′2i.

The learning rate parameter *μ* and the initial values for *v*_0_ and *c* are defined in [19].

Hence, the instantaneous resistance values are calculated over the whole measurement data at any sampling frequency allowing for analysis of dynamic changes in the continuous nasal airflow resistance.

### 2.4. Heart Rate Variability Analysis

At first, the baseline fluctuation of the measured ECG signals was removed with a Savitzky-Golay filter (polynomial order 2). R-peaks were automatically detected from the filtered ECG signals based on the expected time between adjacent R-waves and amplitude threshold. The results were verified visually and corrected manually for false alarms and missed peaks. Tachogram was derived from the R-R intervals and converted to equidistantly sampled series by interpolation, in this case to the frequency of 4 Hz.

The tachogram was filtered with the bandpass Finite Impulse Response (FIR) filters with the cutoff frequencies corresponding to LF and HF bands. The LF band was set between 0.04 Hz and 0.15 Hz and HF band was set between 0.15 Hz and 0.4 Hz, as recommended by the Task Force [14]. The variance, which is the correlate of the signal power, was then computed in 3 min windows from the filtered signals. Thereafter, the window was shifted ahead by 18 sec (overlap of 90%) and the variance was computed again, which produced the LF and HF power signals. Typically, LF component is considered to reflect mostly the sympathetic modulation, HF component is considered to reflect mostly the parasympathetic modulation, and LF/HF ratio is considered to reflect mostly the sympathovagal balance, although this interpretation is not fully agreed among researches [14].

### 2.5. Statistics

Statistical significance of the changes in nasal airflow resistance, LF, HF, and LF/HF ratio and their trends in the subjects was assessed by Wilcoxon signed-rank test. Statistical significance between the subject groups in its turn, was assessed by Mann-Whitney test (Wilcoxon rank-sum test). The null hypothesis was that there are no differences in the medians of given data sets. The results were reported as statistically significant if a two-sided *p* value was less than 0.05. Additionally, logistic regression was used to determine the best factors for predicting whether the subject is birch pollen allergic or not.

## 3. Results

ECG, respiratory effort signals, and pressure were recorded according to the measurement protocol described in Section 2.2. At first, the rib cage and abdominal effort belt signals were calibrated to flow of spirometer from the calibration recording. Then, continuous nasal airflow resistance signals were computed from the nasal pressure and calibrated respiratory effort belt signals. LF, HF, and LF/HF ratio signals were computed from the ECG signal. In this paper, we studied only the third protocol phase, where the allergic reactions for the birch pollen challenge were expected to appear.

Interestingly, it was observed that there is a linear trend in the resistance signals and the HRV signals in all allergic subjects. Thus, linear line fitting was performed to the trends to determine the slopes and their differences. For the allergic subjects, those parts of the nasal airflow resistance curves were selected to the trend analysis, where the resistance increased from the start of the protocol phase until some saturation point. However, individual variation of the time span of the resistance increase was large and for such reason analysis window varied between the subjects.

Importantly, it was observed that the trends in ANS parameters turned out not to be the same in length as trends in resistance. To our knowledge, this is the first time this kind of differences in timing between continuous nasal airflow resistance and ANS function is found out. We therefore selected different time windows for trend analysis for HRV signals in order to guarantee that only linear segments are considered. The starting time for the window was always the same as with the resistance signal for each subject, but the ending time depended on the actual end of the trend.

For the nonallergic subjects, no obvious trends were observed. Thus, the whole nasal airflow resistance, LF, HF, and LF/HF ratio signals from the third protocol phase were chosen to the trend analysis, excluding first the artefacts and the possible nonspecific nasal hyperreactivity at the beginning of the phase.

**Figure 4:**
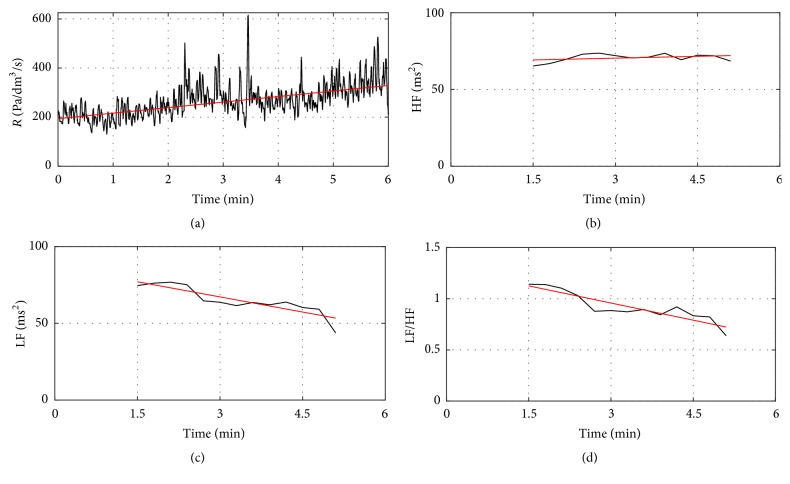
An increase of nasal airflow resistance and related LF, HF, and LF/HF ratio curves for birch pollen allergic subject 1. (a) Resistance curve (black) and robust fit of the resistance curve (red). (c) LF curve (black) and robust fit of the LF curve (red). (b) HF curve (black) and robust fit of the HF curve (red). (d) LF/HF ratio curve (black) and robust fit of the LF/HF ratio curve.

An example case of concomitant dynamic changes in the nasal airflow resistance, LF, HF, and LF/HF ratio curves of birch pollen allergic subject after allergen provocation is shown in Figure 4. A clear upward trend can be observed in the nasal airflow resistance curve and downward trends can be seen in the LF and LF/HF ratio curves. A robust line fitting algorithm was applied to the curves to quantify the extent of slopes.

**Figure 5:**
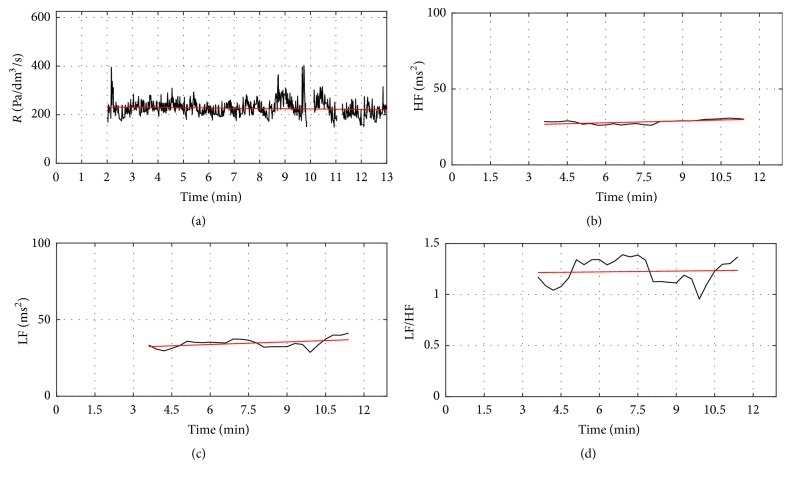
Nasal airflow resistance and related LF, HF, and LF/HF ratio curves for nonallergic subject 3. (a) Resistance curve (black) and robust fit of the resistance curve (red). (c) LF curve (black) and robust fit of the LF curve (red). (b) HF curve (black) and robust fit of the HF curve (red). (d) LF/HF ratio curve (black) and robust fit of the LF/HF ratio curve.

A representative case of concomitant nasal airflow resistance, LF, HF, and LF/HF ratio curves of nonallergic subject after allergen provocation is shown in Figure 5. A gap at the beginning of resistance curve can be seen due to removing of artifacts during manual validation.

Table 1 presents the slopes and changes of nasal airflow resistance, LF, HF, and LF/HF ratio curves for each birch pollen allergic subject, together with their average values and standard deviations (SD) over the subject group. As can be seen from Table 1, LF and LF/HF ratio decreased consistently during the increase of nasal airflow resistance for all the birch pollen allergic subjects (*p* = 0.000), while the HF power changes had both positive and negative slopes. There was a statistically significant change in the resistance (*p* = 0.002), LF (*p* = 0.002), and LF/HF ratio (*p* = 0.002). There was no statistically significant change in HF (*p* = 0.432).

Table 2 presents the slopes and changes of resistance, LF, HF, and LF/HF ratio curves for each nonallergic subject, together with their average values and standard deviations over the subject group. In this group, LF and LF/HF ratio increased for 9/10 subjects. There was only one subject with decreasing LF and one subject with decreasing LF/HF ratio. A noticeable issue is that these two were different subjects and there was no subject with the simultaneous decreasing LF and decreasing LF/HF ratio. There was a statistically significant change in the LF (*p* = 0.037) and LF/HF ratio (*p* = 0.006) and no statistically significant change in resistance (*p* = 0.865) and HF (*p* = 0.432).

Between the groups, a statistically significant difference was found in several parameters, the slope of resistance (*p* < 0.001), resistance at the end (*p* = 0.029), change of resistance (*p* < 0.001), slope of LF (*p* < 0.001), change of LF (*p* < 0.001), slope of LF/HF ratio (*p* < 0.001), and change of LF/HF ratio (*p* < 0.001), between the birch pollen allergic and nonallergic groups. However, there was no statistically significant difference in the resistance at the beginning (*p* = 0.579), slope of HF (*p* = 0.529), and change of HF (*p* = 0.912) between the two groups.

Logistic regression was applied to determine parameters, which predict the allergy status (allergic or nonallergic) of the subjects most reliably. When the used parameter was the slope of LF/HF ratio, the logistic regression model predicted correctly 9/10 birch pollen allergic and 9/10 nonallergic subjects. When the parameter used was the slope of LF, the model predicted correctly 10/10 birch pollen allergic and 9/10 nonallergic subjects. The best result was achieved when both parameters, the slope of LF and the slope of LF/HF ratio, were used, in which case the model predicted correctly 10/10 birch pollen allergic and 10/10 nonallergic subjects.

## 4. Discussion and Future Work

Here, we conducted an experimental trial, where birch pollen allergic subjects and nonallergic control subjects had topical nasal challenge with birch pollen extract. We monitored both the immediate nasal reaction and simultaneous autonomic nervous system function by recording continuous nasal airflow resistance and by analyzing heart rate variability, respectively. We found that in allergic subjects the nasal airflow resistance increased and concomitantly the LF energy and LF/HF ratio decreased after allergen challenge indicating sympathetic withdrawal. In addition, the change in HF energy or the heart rate signal itself did not show similar dynamics. The sympathetic stimulus in the nose is known to cause vasoconstriction and thus increased nasal patency. Thus, our findings indicate that in allergic subjects the nasal obstruction following topical nasal exposure to allergen is at least partially due to the dysfunction of the autonomic nervous system. The LF energy and LF/HF ratio were strong parameters that differentiated the allergy group from the nonallergy group. Additionally, we found out that there are differences in timing between the length of increase in nasal airflow resistance and the length of trend in LF energy and LF/HF ratio signals. This finding was only possible with our proposed technique to estimate continuous nasal airflow resistance and HRV parameters. To our knowledge, this is the first time this kind of differences is found out. Study of meaning and importance of this phenomenon remains as future work.

Our findings are in accordance with those of Pichon et al. who followed subjects with lower respiratory symptoms after diagnostic methacholine bronchial challenge using heart rate variability analysis and found that the hyperresponsive subjects had altered autonomic balance [24]. However, Pichon et al. found that the hyperresponsive subjects had higher parasympathetic tone (HF component) than those without airway responsiveness, both at baseline and after the challenge, whereas we found the decrease of sympathetic tone (LF component) to be responsible for the altered autonomic reaction in the allergic subjects after topical nasal challenge. Our findings are also in accordance with studies by Lan et al. [16], Taşcilar et al. [17], and Yokusoglu et al. [18], who concluded that autonomic dysfunction may play a role in allergic rhinitis.

Measuring nasal reactions after topical nasal challenge is difficult in clinical work. The measurement inevitably affects the delicate nose and easily causes a false reaction. For example, the weighing of nasal secretion after the challenge is done by a suctioning device which irritates the nose and can itself cause nasal obstruction and secretion. Similarly, the measurement of nasal breathing with conventional rhinomanometry or acoustic rhinometry involves occlusion of one nostril that interferes with the normal nasal breathing. These disadvantages can be totally avoided if nasal allergy can be measured from the heart rate without touching the nose at all, like our present results indicate.

The work included a number of limitations. Firstly, the study included only ten birch pollen allergic and ten nonallergic subjects. Still, the sample enabled us to find significant associations between the HRV results and the resistance values and allergy status. The study should be repeated with a larger data set in order to draw more general conclusions. Secondly, it was observed that the timing of ANS response to provocation was not the same as with nasal airflow resistance. We selected signal segments manually for the line fitting procedure. Automating the segment selection would be highly beneficial for practical applications and is left for future work. Thirdly, the window size for HRV analysis was chosen as 3 min for the following reasons. Our data contained strong dynamics after the challenge of birch pollen allergic subjects. Traditional 5 min window was too long for our purposes because the rise of nasal airflow resistance was shorter than 5 min for some allergic subjects. However, due to the dynamics, 1 min window produced very unstable data. The choice of 3 min window was an experimental compromise. However, robustness to dynamic variation and, indeed, analysis of the variation itself still remains as future work.

## 5. Conclusion

We presented a method to assess simultaneous dynamic changes in nasal airflow resistance and autonomic nervous system function during a provocation test. The continuous nasal airflow resistance was estimated with a method that applies LMS filtering technique to the nasal pressure signals and calibrated rib cage and abdominal effort belt signals to update adaptively an extended Broms model. LF and HF components of heart rate were quantified with bandpass FIR filter in 3 min 90% overlapping windows.

Quantitative results of nasal airflow resistance, LF, HF, and LF/HF ratio changes were presented for birch pollen allergic and nonallergic subjects. The main finding was that when the nasal airflow resistance of birch pollen allergic subjects increases after the provocation, the LF energy and LF/HF ratio decrease with a clear trend. This could imply gradual sympathetic withdrawal in allergic patients after the provocation with allergen. Our findings further imply that the allergic and nonallergic subjects could be differentiated by following HRV after the provocation. We also found out that there are differences in timing between the length of increase in nasal airflow resistance and the length of trend in LF energy and LF/HF ratio signals. This finding was only possible with our proposed technique to estimate continuous nasal airflow resistance and HRV parameters. The proposed method opens entirely new opportunities to research accurately concomitant changes in nonstationary nasal breathing function and autonomic nervous system in provocation tests.

## Figures and Tables

**Table 1:** Nasal airflow resistance, LF, HF, and LF/HF ratio results for birch pollen allergic subjects.

Subject	Slope of resistance	Change of resistance [Pa/dm^3^/s]	Slope of LF	Change of LF	Slope of HF	Change of HF	Slope of LF/HF ratio	Change of LF/HF ratio
1	0.0121	135	−259.9	−3118	32.5	390	−0.063	−0.75
2	0.0108	110	−28.6	−1115	−3.9	−51	−0.024	−0.94
3	0.0045	67	−25.0	−300	1.1	13	−0.262	−3.14
4	0.0159	266	−11.4	−262	10.2	235	−0.021	−0.49
5	0.0087	113	−359.8	−1799	60.1	300	−0.238	−1.19
6	0.0087	73	−10.6	−117	22.2	244	−0.016	−0.17
7	0.0132	269	−12.3	−172	0.7	10	−0.041	−0.58
8	0.0019	41	−12.7	−481	1.3	49	−0.075	−2.84
9	0.0037	34	−25.7	−154	−110.2	−661	−0.002	−0.01
10	0.0002	9	−14.2	−798	−0.9	−51	−0.018	−1.03
Average ± SD	0.0080 ± 0.0052	112 ± 91	−76.0 ± 125.6	−832 ± 965	1.3 ± 44.0	48 ± 294	−0.076 ± 0.094	−1.11 ± 1.05

**Table 2:** Nasal airflow resistance, LF, HF, and LF/HF ratio results for nonallergic subjects.

Subject	Slope of resistance	Change of resistance [Pa/dm^3^/s]	Slope of LF	Change of LF	Slope of HF	Change of HF	Slope of LF/HF ratio	Change of LF/HF ratio
1	0.0001	3	17.3	623	0.7	27	0.045	1.62
2	0.0001	4	5.7	262	–1.1	–52	0.024	1.09
3	–0.0005	–11	12.2	318	7.0	181	0.002	0.05
4	0.0005	14	62.3	2805	36.7	1653	0.009	0.40
5	–0.0031	–92	1.7	71	–1.0	–41	0.026	1.09
6	0.0014	33	54.5	1800	57.8	1907	0.018	0.59
7	0.0000	1	20.3	753	14.3	530	–0.005	–0.17
8	0.0004	12	26.9	994	–14.5	–535	0.015	0.56
9	–0.0005	–16	0.4	21	–10.7	–556	0.007	0.36
10	–0.0001	–3	–15.4	–861	–4.4	–245	0.010	0.55
Average ± SD	–0.0002 ± 0.0012	–5 ± 33	18.6 ± 24.2	679 ± 1020	8.5 ± 22.5	287 ± 851	0.015 ± 0.014	0.61 ± 0.53
